# The human parasite *Leishmania amazonensis* downregulates iNOS expression via NF-κB p50/p50 homodimer: role of the PI3K/Akt pathway

**DOI:** 10.1098/rsob.150118

**Published:** 2015-09-23

**Authors:** Teresa C. Calegari-Silva, Áislan C. Vivarini, Marina Miqueline, Guilherme R. R. M. Dos Santos, Karina Luiza Teixeira, Alessandra Mattos Saliba, Simone Nunes de Carvalho, Laís de Carvalho, Ulisses G. Lopes

**Affiliations:** 1 Laboratório de Parasitologia Molecular, Instituto de Biofísica Carlos Chagas Filho, CCS, UFRJ, Rio de Janeiro, Brazil; 2 Laboratório Cultura de Células, Departamento de Histologia e Embriologia, Universidade do Estado do Rio de Janeiro, Rio de Janeiro, Brazil; 3 Departamento de Microbiologia e Parasitologia, Da Faculdade de Ciências Médicas, Universidade do Estado do Rio de Janeiro, Rio de Janeiro, Brazil

**Keywords:** nuclear factor kappa B, *Leishmania*, PI3K/Akt, macrophage

## Abstract

*Leishmania amazonensis* activates the NF-κB transcriptional repressor homodimer (p50/p50) and promotes nitric oxide synthase (iNOS) downregulation. We investigated the role of PI3K/Akt in p50/p50 NF-κB activation and the effect on iNOS expression in *L. amazonensis* infection. The increased occupancy of p50/p50 on the iNOS promoter of infected macrophages was observed and we demonstrated that both p50/p50 NF-κB induction and iNOS downregulation in infected macrophages depended on PI3K/Akt activation. Importantly, the intracellular growth of the parasite was also impaired during PI3K/Akt signalling inhibition and in macrophages knocked-down for Akt 1 expression. It was also observed that the increased nuclear levels of p50/p50 in *L. amazonensis*-infected macrophages were associated with reduced phosphorylation of *907 Ser* p105, the precursor of p50. Corroborating these data, we demonstrated the increased levels of phospho-*9 Ser* GSK3β in infected macrophages, which is associated with GSK3β inhibition and, consequently, its inability to phosphorylate p105. Remarkably, we found that the levels of pPTEN *370 Ser*, a negative regulator of PI3K, increased due to *L. amazonensis* infection. Our data support the notion that PI3K/Akt activity is sustained during the parasite infection, leading to NF-κB 105 phosphorylation and further processing to originate p50/p50 homodimers and the consequent downregulation of iNOS expression.

## 1. Introduction

The transcription factor nuclear factor kappa B (NF-κB) regulates the expression of a number of immunological mediators, including the chemokines, cytokines, adhesion molecules and enzymes that produce secondary inflammatory mediators such as nitric oxide synthase (iNOS) [1]. The NF-κB family comprises five different members containing the Rel homology domain that may originate homo- or heterodimers, NF-κB1 (p105/p50), NF-κB2 (p100/p52), RelA (p65), RelB and c-Rel [2]. NF-κB1 and NF-κB2 are synthesized as the large p105 and p100 precursors, which are post-translationally processed to the DNA-binding subunits p50 and p52, respectively [3]. The canonical activation of NF-κB involves the phosphorylation of IκB (NF-κB inhibitor) by the IκB kinase (IKK) complex, leading to proteasome-mediate IκB degradation and nuclear translocation of NF-κB dimer RelA–p50 [4]. NF-κB is frequently activated during infections and plays an important role in initiating innate immune responses [5,6]. Conversely, the alternative pathway involves the nuclear translocation of RelB heterodimers and requires the phosphorylation and proteasome processing of the precursor protein p100, which originates the subunit p52. Activation of the alternative pathway is triggered by specific stimuli such as LT*β*R [7] and is important for the development of lymph nodes. The controlling mechanisms involved in the generation and dynamics of distinct NF-κB dimers is still poorly understood so that a number of theoretical and experimental approaches have been developed to address this issue [8].

Sandfly vectors transmit infective metacyclic promastigotes to vertebrates. In vertebrate hosts, promastigotes differentiate to amastigotes inside parasithophorous vacuoles [9,10]. Leishmaniasis affects around 12 million people worldwide with approximately 2 million new infections per year (WHO/TDR (World Health Organization/Tropical Diseases Researchers); http://www.who.int/tdr/diseases/leish/direction.htm#burden).

*Leishmania* parasites exhibit a plethora of parasitic life adaptive mechanisms and are particularly effective in escaping the host immune response. Several reports have indicated that *Leishmania* both interferes with signal transduction pathways and alters the balance between the microbicidal and suppressor functions exhibited by macrophages [11–17].

*Leishmania amazonensis* infections are characterized by the suppression of the inherent initial response, noted by inhibition of macrophage production of pro-inflammatory molecules. It has been shown that in the initial days of infection by *L. amazonensis,* some inflammatory cytokines are downregulated when compared with *L. major* infected mice [18]. Another study reported the suppression of pro-inflammatory molecules (IL-12, IL-17 and IL-6) in macrophages infected with *L. amazonensis* and treated with lipopolysaccharide (LPS) as compared with infection by *L. major* [19].

We have demonstrated the activation of NF-κB transcriptional repressor homodimer (p50/p50) in *L. amazonensis-*infected macrophages and the downregulation of iNOS expression in infected macrophages associated with the p50/p50 formation [13].

Processing of p105 precursor is required for the generation of p50 units. The stability of the p105 subunit requires the phosphorylation of the 907 serine residue, which, mediated by glycogen synthase kinase 3 beta (GSK3β) in resting cells, prevents the constitutive processing of p105 to p50 and stabilizes p105, leading to proteolytic processing in response to TNF-α [20]. The enzyme Akt inactivates GSK3β through the phosphorylation of serine 9 [21] and thereby regulates p105 processing to generate the p50 subunit. The objective of this work was to investigate the role of PI3K/Akt in p50/p50 NF-κB activation and iNOS downregulation in *L. amazonensis* infection.

## 2. Material and methods

### 2.1. Cell culture

The human monocytic leukaemia cell line THP-1 (ATCC: TIB202TM) was cultured in RPMI (Gibco) medium supplemented with 10% fetal bovine serum (Sigma), 1 mM pyruvate, 200 mM l-glutamine, 100 U ml^−1^ penicillin and 100 mg ml^−1^ streptomycin in an incubator at 37°C with 5% CO_2_. These cells were differentiated to macrophages with 40 ng ml^−1^ PMA (Sigma) for 72 h. Afterwards, the cells were washed with PBS and incubated with fresh medium for more than 72 h. Mouse macrophage leukaemia cell line RAW 264.7 (ATCC: TIB-71) and human embryonic kidney cell line HEK-293FT (Life Technologies) were cultured in DMEM (Gibco) medium supplemented with 10% fetal bovine serum, 100 U ml^−1^ penicillin and 100 mg ml^−1^ streptomycin in an incubator at 37°C with 5% CO_2_. HEK-293FT cells were maintained in medium containing 500 µg ml^−1^ Geneticin.

### 2.2. Murine primary macrophages

Thioglycollate-elicited peritoneal macrophages were removed from C57BL/6 mice by peritoneal washing and enriched by plastic adherence onto 6-well polystyrene plates (2 × 10^6^ per well, 1 h at 37°C). Non-adherent cells were washed out with PBS, and the adherent cell population was incubated for 24 h in DMEM with 10% fetal bovine serum for subsequent *Leishmania* infection assays.

### 2.3. Primary human macrophages

Monocyte-derived macrophages were obtained from peripheral blood mononuclear cells (PBMCs) isolated from buffy coat preparations of human healthy blood donors as previously described [22].

### 2.4. Parasites, culture conditions and infection

*Leishmania amazonensis* (WHOM/R/75/Josefa) was maintained *in vitro* in Schneider Insect Medium (Sigma) supplemented with 10% fetal bovine serum. Promastigotes were passed to fresh medium when the cells reached the density of 10^7^ parasites ml^−1^, at 26°C. Macrophages were infected with promastigotes collected at the stationary phase 4–5 days after inoculation of the culture at a parasite-to-cell ratio of 5 : 1. The infection index was calculated by multiplying the percentage of infected macrophages by the average number of parasites per macrophage on Giemsa-stained slides.

### 2.5. Cell treatment

Cells were treated with 1 µg ml^−1^ of LPS (Sigma-Aldrich). To inhibit the PI3K/Akt pathway, cells were treated with 10 µM of LY294002 (Sigma-Aldrich) or 1 µM of wortmannin (Sigma-Aldrich) or 5 µM of Akt inhibitor VIII, isozyme-selective, Akti-1/2 (Santa Cruz Biotechnology) during the infection.

### 2.6. Electrophoretic mobility assay

Differentiated THP-1 (4 × 10^6^ cells) was infected and the nuclear extracts obtained and submitted to electrophoretic mobility assay (EMSA), as previously described [13].

### 2.7. Immunofluorescence

Differentiated THP-1 (2 × 10^5^ cells) was infected and fixed in 4% paraformaldehyde and processed for immunofluorescence as follows: after incubation with ammonium chloride and 0.5% Triton X-100 solution in PBS, cells were blocked with 5% bovine serum albumin (BSA, Sigma-Aldrich) in PBS solution and incubated with anti-rabbit p50 polyclonal antibody (Millipore—06-886) overnight, followed by incubation with Alexa 555-conjugated anti-rabbit secondary antibody (Life Technologies). Cells were then stained with DAPI nuclear dye (Sigma-Aldrich) and mounted in ProLong Gold anti-fade media (Life Technologies) for further analyses in an LSM META 510 (Carl Zeiss, Germany) laser scanning confocal microscope.

### 2.8. Luciferase assays

To investigate the NF-κB-dependent transcriptional activity, RAW 264.7 cells (2 × 10^5^) were seeded onto 24-well plates and transfected using Lipofectamine 2000 reagent (Invitrogen). For the transfections, 2 µg p6κB-LUC (kindly provided by Dr Patrick Baeuerle) and 80 ng pRL-CMV (Promega) were used. Cells transfected with p6κB-LUC plasmids were infected and treated with LPS (1 µg ml^−1^, Sigma). Subsequent to infection and treatment, the cells were washed with PBS, lysed according to Dual Luciferase System protocol (Promega) and analysed in a TD-20/20 Luminometer (Turner Designs).

### 2.9. Quantitative real-time PCR

Total RNA of RAW 264.7 cells (3 × 10^6^) was extracted with RNeasy® Plus (Quiagen), and 1 µg aliquot of total RNA was reverse-transcribed into the first-strand cDNA with ImProm (Promega) and oligo(dT) 12–18 primer, according to the manufacturer's instructions. The following pairs of primers were used to determinate mRNA iNOS levels: forward 5′- CAGCTGGGCTGTACAAACCTT-3′ and reverse 5′-CATTGGAAGTGAAGCGTTTCG-3′. GAPDH forward 5′-TGCACCACCACCTGCTTAGC- 3′ and GAPDH reverse 5′-GGCATGGACTGTGGTCATGAG-3′ were used for normalization. The amplicon specificity was carefully verified by the presence of a single melting temperature peak in dissociation curves run after real-time PCR. The detection of a single band of the expected size was verified by electrophoresis. Real-time quantitative PCR (qPCR) was carried out via the Applied Biosystems 7500 detection system using Power SYBR Green PCR Master Mix (Applied Biosystems). All qPCR experiments were performed at least three times. All expression ratios were computed via the ΔΔCt method.

### 2.10. Immunoblotting

Differentiated THP-1 cells were infected with *L. amazonensis*, and total and nuclear extract proteins were obtained [13]. Both nuclear (10 µg) and total proteins (30 µg) were subjected to electrophoresis in 10% SDS-polyacrylamide gels. The proteins were transferred to a nitrocellulose membrane (Amersham). Blots were separately incubated with primary antibody against p50 (Millipore—06-886), lamin A/C (Cell Signalling 2032), phospho105 (serine 907) (sc101746), phosphoGSK3β (Cell Signalling 9326S), GSK3β (Cell Signalling 931S), phosphoPTEN (Serine 370) (GenScript A00290), Akt (Cell Signalling 9272S) and β-actin (Sigma-Aldrich A2228). Antibody anti-rabbit or anti-mouse horseradish peroxidase-conjugated IgG (1 : 3000) was used. The membranes were then submitted to three washings with TBST, and proteins were detected by the ECL chemiluminescent detection system (Amersham Biosciences).

### 2.11. Chromatin immunoprecipitation assay

Chromatin immunoprecipitation (ChIP) analysis was carried out according to the Simple ChIP Enzymatic Chromatin IP Kit protocol (Cell Signalling). After infection and treatment, RAW 264.7 cells (4 × 10^7^) were submitted to ChIP assay as described [17]. The chromatin was immunoprecipitated with anti-p50 (Millipore—06-886) and anti-p65 (sc 372X) antibodies at 4°C under rotation for 16 h. The DNA isolated from immunoprecipitated material was amplified by real-time PCR using SyberGreen for iNOS promoter (−48 to −209): forward 5′-ACACAGACTAGGAGTGTCCATCATGA-3′ and reverse 5′- ACAAGACCCAAGCGTGAGAGGCCTCA- 3′. As a control, 1/50 of digested input chromatin was similarly processed and analysed in the absence of immunoprecipitation.

### 2.12. Lentivirus transduction

THP-1 cell knock-down for Akt 1 expression was obtained by lentivirus transduction as described previously [23]. The human shRNA Akt 1 constructions (shRNA 1—TRC N0000010162; shRNA 2—TCRN0000221552) from Broad Institute, MA, USA were purchased from Sigma-Aldrich. The pMD2.G envelope plasmid, PSPAX2 packaging plasmid and pLVTHM plasmid were kindly provided by Dr. Bertal H. Aktas, Harvard Medical School, MA, USA. After 6 days of transduction, THP-1 cells were selected with 1 µg ml^−1^ of puromycin for 12 days.

### 2.13. Statistical analyses

Data were analysed by Student's *t*-test for independent samples or one-way ANOVA using Prism 5 software. Data are expressed as the average of three independent experimental determinations, and significant differences are indicated for *p* < 0.05.

## 3. Results

### 3.1. *Leishmania amazonensis* increases nuclear levels of p50 NF-κB and promotes its occupancy on iNOS promoter of infected macrophages

**Figure 1.**
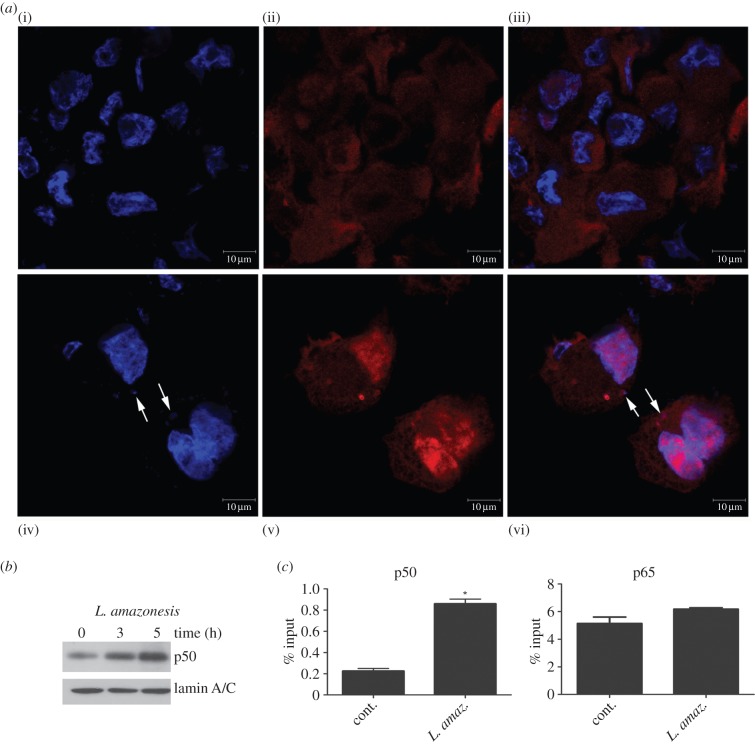
*Leishmania amazonensis* increases NF-κB/p50 subunit nuclear levels and the occupancy on the iNOS promoter. THP-1 cells were differentiated into macrophages and infected with promastigotes of *L. amazonensis* at the indicated times. (*a*) Confocal photomicrographs from control (i)*–*(iii)) and *L. amazonensis* 5 h infection (iv)*–*(vi)) immunostained with p50 (red) and stained with DAPI (blue), using 100× objective (bar = 10 µm). Nuclei stained with DAPI are seen in (i) and (iv). Protein p50 expression in the same cells is observed in (ii) and (v). Figures (iii) and (vi) correspond to merged images showing p50 localization in cytoplasm and nucleus. Cells infected with *L. amazonensis* for 5 h display higher expression of p50 in nuclei (arrows = intracellular *Leishmania amazonensis* stained with DAPI. (*b*) Nuclear protein lysates were obtained and submitted to western blot using anti-p50 specific antibody or anti-lamin A/C. (*c*) RAW 264.7 cells were infected for 5 h, and the ChIP assay was performed with the anti-p50 and anti-p65 antibodies. The immunoprecipitated chromatin was amplified by real-time PCR using specific primers to κB-binding sites in the iNOS gene promoter. *p < 0.05.

To verify whether the nuclear levels of p50 were augmented in infected macrophages, immunofluorescence assay demonstrated the nuclear translocation of p50 after the infection of macrophages with *L. amazonensis* for 1 h (data not shown) and 5 h (figure 1*a*). The immunoblot analysis of nuclear extracts derived from infected macrophages revealed an increase of p50 levels (figure 1*b*), thus corroborating the previous observations.

We decided to test whether the iNOS promoter would be occupied by the induced p50/p50 homodimers. ChIP assays with the chromatin of *L. amazonensis-*infected cells were performed using anti-p50 or anti-p65 antibodies. Corroborating the notion of the importance of p50 homodimers in reducing iNOS expression, as previously reported [13], the increased occupancy of p50 NF-κB on the iNOS promoter of infected macrophages was confirmed. Accordingly, immunoprecipitation with anti-p65 did not reveal any significant changes related to the infection (figure 1*c*).

### 3.2. The p50/p50 iNOS promoter occupancy is maintained in *Leishmania amazonensis*-infected macrophages treated with LPS

**Figure 2.**
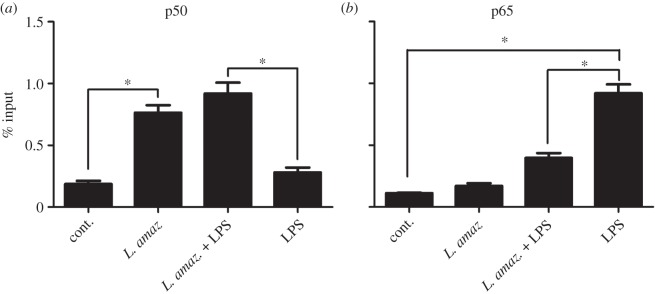
*Leishmania amazonensis* induces p50 NF-κB occupancy on the iNOS promoter in infected macrophages treated with LPS. RAW 264.7 cells were infected with promastigotes of *L. amazonensis* for 5 h and treated with 1 µg ml^−1^ LPS for 1 h. The ChIP assay was performed using (*a*) anti-p50 and (*b*) anti-p65 antibodies. The immunoprecipitated chromatin was amplified by real-time PCR using primers described in the Material and methods section. *p < 0.05.

To evaluate the dynamics of the p50/p50 NF-κB complex in *L. amazonensis-*infected macrophages challenged with LPS, an inducer of the canonical NF-κB heterodimer p65/p50, further ChIP assays were conducted. As predicted, the occupation of the p50/p50 homodimer at Nκ-sites in the iNOS promoter region in LPS-treated and infected macrophages was augmented (figure 2). The p65 immunoprecipitated chromatin exhibited increased occupancy only in the LPS-treated samples (figure 2). These results corroborated previous data [13] suggesting that, in EMSA assays, the LPS-activated complex (p65/p50) is replaced by the *L. amazonensis-*activated complex (p50/p50). These observations are in accordance with the reduced iNOS mRNA and nitric oxide levels observed in *L. amazonensis*-infected macrophages treated with LPS.

### 3.3. *Leishmania amazonensis* induces p50/p50 NF-κB activation through PI3K/Akt pathway

**Figure 3.**
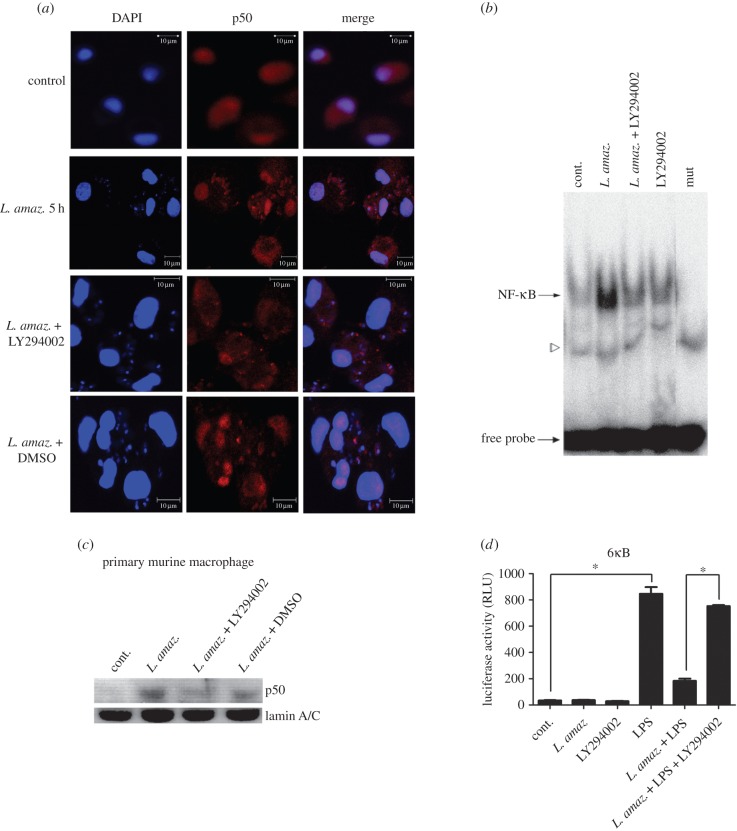
The PI3K/Akt pathway is involved in NF-κB activation during *L. amazonensis* infection. (*a*) Confocal photomicrographs from the infection conditions indicated immunostained with p50 (red) and nuclei stained with DAPI (blue), using 100× objective (bar = 10 µm). Primary human macrophages infected with *L. amazonensis* for 5 h display higher expression of p50 in nuclei. (*b*) Macrophages were infected with promastigotes of *L. amazonensis* and treated with LY 294002 for 1 h. Nuclear extracts were obtained and subjected to EMSA as indicated; mut, NF-κB mutant oligonucleotide. The specific bands of NF-κB are indicated by arrows and a free probe is indicated by the bottom arrow. (*c*) Nuclear protein lysates were obtained from primary murine macrophages and submitted to western blot using anti-p50 specific antibody or anti-lamin A/C. (*d*) RAW 264.7 cells were transiently transfected with the reporter plasmid containing the luciferase gene under the control of six NF-κB consensus binding sites. Twenty-four hours after transfection, the cells were infected and/or treated as indicated. After 24 h, the total protein lysate was analysed for Renilla normalized luciferase activity. RLU, luciferase relative unit. *p < 0.05.

We further approached the role of the PI3K/Akt pathway in the processing of p105 to generate p50 subunits in the context of *L. amazonensis* infection. As previously described [15], *L. amazonensis* was able to activate PI3K/Akt signalling through Akt phosphorylation (473 serine residue) in infected macrophages. To address whether PI3K activity was required for p50/p50 *L. amazonensis* induction, we employed distinct technical approaches. In immunofluorescence assays, we verified that the nuclear translocation of p50 NF-κB subunit was impaired in human primary macrophages infected with *L. amazonensis* and treated with the PI3K inhibitor LY294002, while the nuclear levels of the p65 NF-κB subunit remained unchanged (electronic supplementary material, figure S1). Accordingly, EMSA assay carried out with nuclear extracts of differentiated THP-1 corroborated this data, showing a reduced binding of the NF-κB p50 to the Nκ consensus probe (figure 3*b*). A similar result was obtained by western blot with nuclear extracts of primary murine macrophages infected with *Leishmania* and treated with the pharmacological inhibitor LY294002. It can be observed in figure 3*c* that LY294002 treatment reduced the nuclear p50 translocation.

We then addressed the question whether downregulation of NF-κB-driven expression required PI3K activity. The experimental model consisted of NF-κB gene reporter assays carried out in infected macrophages treated or not treated with LPS. The day after transfection, macrophages were infected with *L. amazonensis* and treated with the PI3K inhibitor LY294002 for 1 h followed by the addition of LPS. After 24 h, luciferase activity was measured. As shown in figure 3*d*, *L. amazonensis* infection reduced the LPS-induced transcriptional activity. However, this reduction was prevented by the inhibition of PI3K. Taken together, these data suggest that PI3K/Akt is required for p50/p50 NF-κB activation and that PI3K inhibition prevents the transcriptional repression driven by NF-κB in LPS-treated infected macrophages**.**

### 3.4. The occupancy of p50/p50 NF-κB on the iNOS promoter led by *Leishmania amazonensis* infection depends on PI3K

**Figure 4.**
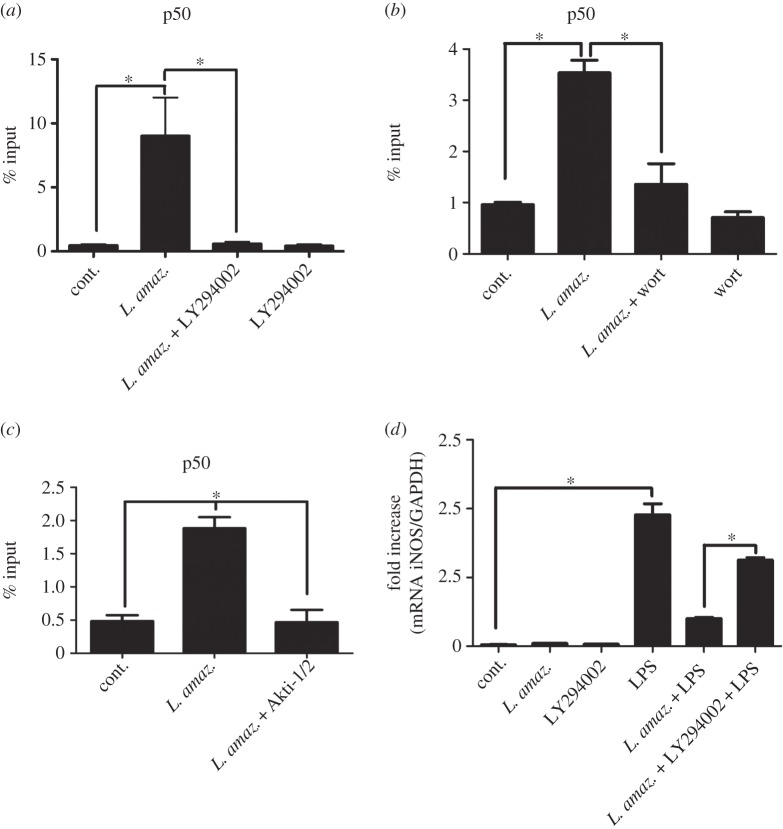
PI3K inhibition reduces p50/p50 occupancy on the iNOS promoter and increases iNOS message levels in infected macrophages. (*a*) RAW 264.7 cells were infected for 5 h with promastigotes of *L. amazonensis* and treated with (*a*) 10 µM LY294002 (PI3K inhibitor), (*b*) 1 µM wortmannin or (*c*) 5 µM Akti-1/2. The ChIP assay was carried out using anti-p50 antibodies. (*d*) Peritoneal macrophages were infected with *L. amazonensis* and/or treated with the PI3K inhibitor LY294002 and/or LPS (1 µg ml^−1^). The samples were subjected to qPCR, as previously described. *p < 0.05.

To verify if the increased p50 occupancy on the iNOS promoter during *L. amazonensis* infection was PI3K-dependent, ChIP assays were performed on RAW 264.7 *L. amazonensis*-infected cells treated with the PI3K inhibitor LY294002. Accordingly, the increased p50 occupancy on the iNOS promoter led by *L. amazonensis* (as shown in figure 1*c*) was abolished in LY294002-treated macrophages (figure 4*a*). Similar results were found when another PI3K inhibitor (wortmannin) or Akt inhibitor VIII, isozyme-selective (Akti-1/2) was used in the experiments (figure 4*b,c*, respectively).

We further investigated the iNOS mRNA levels in *L. amazonensis-*infected macrophages treated with LY294002 and challenged with LPS. As was previously observed, *L. amazonensis* led to a reduction of LPS-induced iNOS mRNA levels. However, PI3K inhibition significantly prevented this effect (figure 4*d*).

### 3.5. *Leishmania amazonensis* modulates PI3K/Akt effectors, thus favouring p50/p50 NF-κB formation

**Figure 5.**
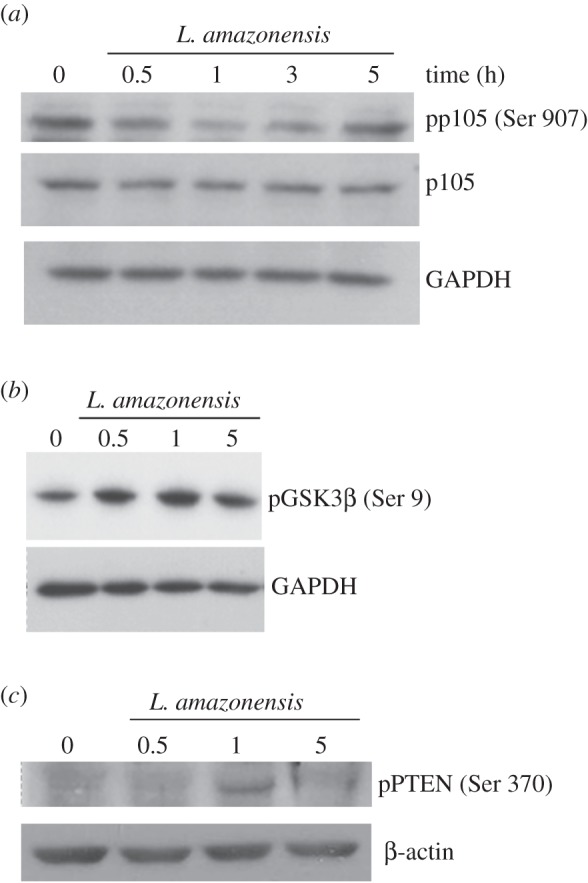
*Leishmania amazonensis* reduces the phosphorylation levels of p105 through inhibition of GSK3β and inactive PTEN in infected macrophages. THP-1 cells were differentiated into macrophages and infected with promastigotes of *L. amazonensis*, as indicated. The protein extracts were obtained and subjected to Western blot using (*a*) the anti-p105 antibodies phosphorylated at serine 907 residue and anti-p105, (*b*) anti-GSK3β phosphorylated at serine 9 residue and (*c*) anti-PTEN phosphorylated at serine 370 residue. Anti-β-actin and anti-lamin A/C were used as loading control.

The p105 phosphorylation of Ser 907 is associated with stability of p105 and further processing. A western blot assay was performed by using a total extract of differentiated THP-1 cells infected with *L. amazonensis*. Figure 5*a* shows the transient reduction of p105 phosphorylated at Ser 907 at 30 min, 1 and 2 h of infection. These data suggest that p105 under these conditions is transiently stable and prone to further processing to generate p50 subunits.

Because the phosphorylation of p105 at Ser 907 is GSK3β mediated, the next step was to determine the phosphorylation levels of this kinase during *L. amazonensis* infection. The inactivation of this enzyme is Akt-mediated by direct phosphorylation at Ser 9 [21,24]. Figure 5*b* shows increased levels of phospho GSK3β in the extracts of infected macrophages.

PTEN (phosphatase and tensin homologue) phosphorylation levels were also verified. This phosphatase is a negative regulator of PI3K due to the conversion of PIP3 (phosphatidyl-inositol-3-phosphate) into PI2P (phosphatidyl-inositol-2-phosphate), which prevents accumulation of PIP3 and the consequent activation of Akt [25]. The Ser 370 phosphorylation of PTEN impairs the phosphatase activity yet maintains the stability of this protein [24,26]. Increased PTEN phosphorylation levels were observed within 1 h of infection (figure 5*c*). Taken together, these data indicate that *L. amazonensis* reduces p105 phosphorylation levels through GSK3β inhibition due to inactive PTEN in infected macrophages, which, in turn, favours the maintenance of PI3K pathway activation.

### 3.6. Impaired p50/p50 NF-κB activation in Akt 1 knocked-down macrophages infected with *Leishmania amazonensis*

**Figure 6.**
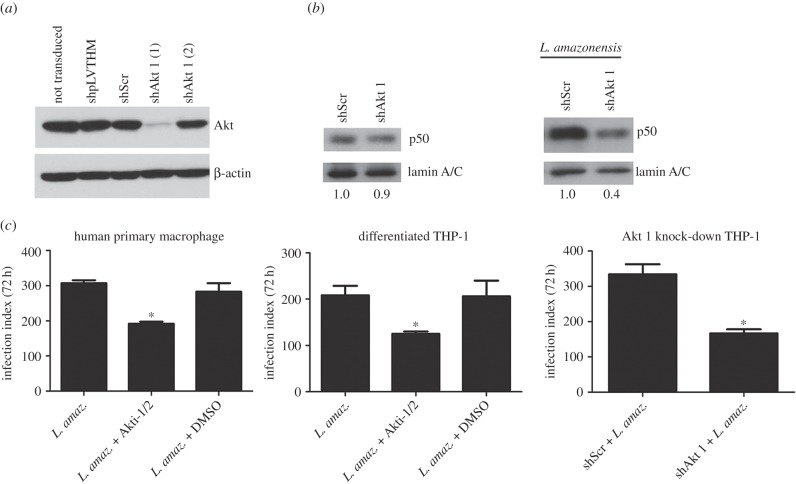
Akt 1 knocked-down expression impairs NF-κB-p50 activation in infected macrophages. (*a*) Western blot of total protein extract obtained from differentiated THP-1 stably knocked-down for Akt 1 expression. Monocytic THP-1 were not transduced or transduced with lentivirus carrying the construction: shpLVTHM, shScr (Scrambled), shAkt 1 (1) and shAkt 1 (2). (*b*) Western blot of nuclear protein extract from differentiated THP-1 knocked-down for Akt 1 (shAkt 1 (1)) and infected with *L. amazonensis* for 5 h. Relative levels of nuclear p50, calculated by densitometry and normalized with the endogenous control lamin A/C, are indicated at bottom. (*c*) Human primary macrophages and differentiated THP-1 were infected with metacyclic promastigote of *L. amazonensis* and treated with Akt 1/2 inhibitor. Differentiated THP-1 knocked-down for Akt 1 was infected with *L. amazonensis* metacyclic promastigote. After 72 h, the infection index was evaluated as described in Material and methods. *p < 0.05.

To definitely demonstrate the requirement of Akt 1 in the induction of p50 NF-κB due to *L. amazonensis* infection, we successfully stably knocked down Akt 1 in THP-1 cells throughout shRNA lentivirus transduction using the shAkt 1(1) construction (figure 6*a*). Nuclear protein extracts of Akt 1 silenced macrophages infected with *L. amazonensis* were obtained and submitted to western blot. Figure 6*b* shows a reduction of the nuclear translocation of p50 subunit due to infection in Akt 1 knocked-down cell extracts.

Our next aim was to evaluate the impact of Akt 1 silencing or drug inhibition on the replication of *Leishmania* in macrophages*.* Human primary macrophages or differentiated THP-1 cells were infected with stationary-phase metacyclic *L. amazonensis* promastigotes. The effect of PI3K/Akt 1 inhibitors on parasite entrance and replication was further assessed. There was no significant change observed in the parasite internalization in 4 h of infection in cells treated with Akt 1/2 inhibitor or LY294002, *p > 0.05 (electronic supplementary material, figure S2*a*). However, at 72 h post-infection Akt 1 inhibition caused a marked decrease in the infection index in Giemsa-stained cells (figure 6). A similar effect was observed when LY294002 was used (electronic supplementary material, figure S2*b*). Importantly, the infection of Akt 1 silenced macrophages also showed reduced *Leishmania* load (figure 6). These data suggest that PI3K and Akt 1 signalling is important for the intracellular growth of the parasite.

## 4. Discussion

The adaptive fitness of intracellular parasites has evolved with the development of sophisticated mechanisms of escape from microbicidal pathways. The host-cell invasion by pathogens often induces transcription factor NF-κB activation, which plays an important role in the initiation of the innate immune response by regulating the expression of many immune mediators, including chemokines, cytokines, adhesion molecules and enzymes that produce such secondary inflammatory mediators as iNOS [3]. *Leishmania* parasites have the ability to survive and multiply inside macrophages by altering signalling cascades and subverting their leishmanicidal functions. Several studies have investigated the modulation of different host-signalling pathways associated with *Leishmania* infection [11,12,27], including signalling via NF-κB [28–34].

The activation of the canonical transcriptional NF-κB heterodimer RelA/p50 has been widely reported in several infections [35]. The parasite *L. amazonensis* induces the repression of the initial macrophage response and is also associated with several degrees of downregulation of the host immunity [18,19]. Our study has demonstrated that *L. amazonensis* activates the transcription repressor NF-κB homodimer p50/p50, primarily associated with downregulation of iNOS expression in infected macrophages even when the cells are treated with external inducers such as LPS [13].

In this work, the mechanisms involved in p50/p50 NF-κB activation in addition to the transcriptional repression of the iNOS gene in *L.* amazonensis-infected macrophages were unveiled. Initially, increased nuclear p50 NF-κB levels (figure 1*a,b*) as a result of infection were observed. Through ChiP assays, it was possible to verify an augmented occupancy of the p50/p50 NF-κB complex at the iNOS promoter (figure 1*c*). However, in the infected macrophages the occupation of the p65 NF-κB subunit at this site remained unaltered (figure 1*c*). Surprisingly, the high occupancy of p50/p50 homodimers in the iNOS promoter region continued to prevail despite the presence of pro-inflammatory inducers such as LPS. These data further strengthen our previous observations that *L. amazonensis* seems to either replace or prevent the onset of the LPS-activated p65/p50 NF-κB complex, leading to p50/p50 subunit binding to the iNOS promoter [13].

As the PI3K/Akt pathway may be involved in p105 NF-κB subunit processing and hence the formation of the p50 subunit, the role of this pathway in the activation of the p50/p50 NF-κB complex as well as the expression of iNOS downregulation in *L.* amazonensis-infected macrophages was evaluated. Several pathogens including viruses and bacteria are able to modulate the PI3K/Akt pathway during infection [36,37]. It has recently been shown that the activation of this pathway in macrophages infected with *L. major*, *L. pifanoi* or *L. amazonensis* resulted in apoptosis resistance of the host cell [14]. It has also been reported that activation of the PI3K/Akt pathway in *L. amazonensis*-infected macrophages negatively affects IL-12 production [15]. Oghumu & Satoskar [37] recently showed that the use of the PI3Kγ inhibitor for the treatment of experimental *L. mexicana* infection in mice resulted in significantly lower parasite burdens and lesion sizes than in wild-type untreated mice.

Our data indicate that the p50/p50 NF-κB activation induced during *L. amazonensis* infection depends on PI3K/Akt activation (figure 3*a–c*). Additionally, gene reporter assays using a construct containing consensus Nκ-sites revealed that the repression of NF-κB promoter activity in LPS-treated and infected macrophages relies on PI3K activation (figure 3*d*). The effect of the PI3kγ-specific inhibitor was also evaluated in our model, but we did not observe any impairment in NF-κB p50 translocation to the nuclei of infected macrophages.

Previous results have suggested that iNOS downregulation of expression in *L. amazonensis-*infected macrophages is due to p50/p50 NF-κB activation. Analysis of the iNOS promoter through ChiP assays has demonstrated that the increased occupation of the p50/p50 homodimer binding site for NF-κB in infected macrophages stems from PI3K/Akt activation (figure 4*a*). We also verified the same effect using other PIK3 K/Akt inhibitors (figure 4*c,d*). Corroborating these data, we have found that iNOS messenger levels in infected macrophages treated with LPS increased during inhibition of PI3K (figure 4*b*).

As described, phosphorylated p105 levels may dictate its destiny to degradation or p50 subunit processing. The phosphorylation levels, in turn, can be changed as a result of PI3K/Akt pathway activation [20]. In our experiments, it were observed increased GSK3β phosphorylation levels at the serine 9 residue (figure 5*b*), which probably was mediated by Akt, leading to its inhibition. Consistent with this observation, it was found that the p105 levels phosphorylated at the serine 907 residue were reduced at the early stage of infection (figure 5*a*). These results imply that the *L. amazonensis-*infected macrophage inhibits GSK3β via Akt, leading to reduced levels of the phosphorylated p105 subunit, favouring its processing. PI3K activation was also observed in *L. donovani-*infected macrophages in which GSK3β was inhibited. As a consequence, these macrophages showed increased binding of CREB to the DNA, terminating in IL-10 induced expression [38]. Such regulation may also extend to *L. amazonensis* infections as this species is capable of increasing IL-10 expression in macrophages via PKR activation, which, in response to signs of stress, may activate PI3K/Akt [16,39].

The involvement of PTEN, a PI3K inhibitor, was also found in our work. In its inactive state, PTEN is phosphorylated within a cluster of serine and threonine residues located in the C-terminal domain, including: Thr 366, Ser 370, Ser 380, Thr 382, Thr 383 and Ser 385 [40]. These changes are related to the maintenance of stability because the dephosphorylation of these residues ‘opens’ their phosphatase/phosphatase domain, increasing its activity and promoting its degradation via proteosome [41]. It was also found that the levels of PTEN phosphorylated at the Ser 370 residue increased in *L. amazonensis-*infected macrophages, leading to inhibition of phosphatase activity (figure 5*c*). As a result, PIP3 substrates accumulate, thus favouring the maintenance of PI3K/Akt activation in infected macrophages.

The present authors and others [13,33] have described that infection by *L. mexicana* or *L. amazonensis* results in the prompt proteolytic digestion of RelA. Basak *et al.* [8] formulated models related to the functional relationships in NF-κB signalling and provide compelling evidence that the absence or overexpression of some NF-κB subunits interferes with the dynamic generation of multiple NF-κB dimers. It is conceivable that the RelA processing taking place in *L. amazonensis* infections contributes the balance of free p50 subunits, which, together with the generation of p50 as a consequence of PI3K activation, may result in the sustained formation of p50/p50 and the ensuing repression and specificity of the innate response to infection.

The importance of the PI3K/Akt pathway for the growth of the parasite was demonstrated using pharmacological inhibitors and in Akt 1 silenced human macrophages (figure 6*c*). Moreover, we demonstrated a reduced p50 nuclear translocation during the infection in THP-1 with Akt knock-down (figure 6*b*).

**Figure 7.**
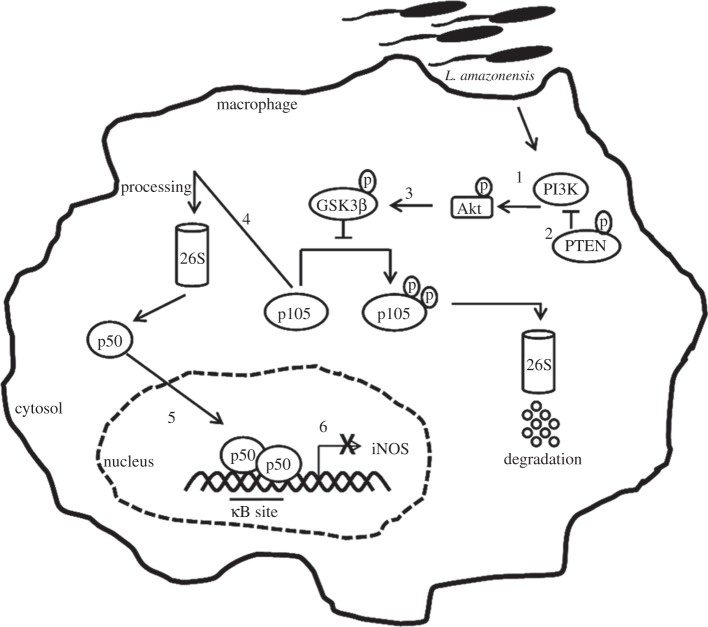
Proposed model for the PI3K/Akt pathway in p50/p50 NF-κB activation in *L. amazonensis* infection. *Leishmania amazonensis* activates PI3K/Akt during the initial period of infection through Akt phosphorylation (1). Sustained activation is favoured by the inhibition of the phosphatase PTEN, the negative regulator of PI3K (2). Akt can phosphorylate GSK3β at serine 9, which is associated with its inhibition. Our model proposes that such an event is happening in *L. amazonensis* infections (3). In uninfected cells, GSK3β is constitutively active and promotes the phosphorylation of p105 at the serine 907 residue, targeting it for degradation. The inhibition of this kinase during *L. amazonensis* infection decreases the levels of phosphorylation at this residue and favours the processing of p105 into a p50 subunit (4). The infection leads to increased p50 nuclear levels (5). We found that iNOS is downregulated in infected macrophages and/or stimulated with LPS due to the p50/p50 complexes that bind to the promoter of this gene. Our work also proposes that the increased occupancy of p50/p50 on the iNOS promoter is PI3K/Akt dependent (6).

In summary, our data show a novel escape mechanism related to the subversion by *L. amazonensis* of NF-κB signalling. We addressed the importance of the PI3K/Akt pathway for the p50/p50 NF-κB complex activation induced by *L. amazonensis* (figure 7). Moreover, we have demonstrated that the iNOS downregulation of expression is due to the p50/p50 NF-κB repressor complex.

Overall, our study has provided evidence that the modulation of the NF-κB pathway is carried out by a species of parasite involved in the repression of macrophage functions. Our findings may serve as a basis for the development of novel drug targets directed to infection by *L. amazonensis*.

## Supplementary Material

p65 NF-kappaB subunit is not involved in NF-kappaB signaling due to L. amazonensis infectio. PI3k/Akt inhibition affects L. amazonensis infection
