# Supplementary material for: The human parasite Leishmania amazonensis downregulates iNOS expression via NF-κB p50/p50 homodimer: role of the PI3K/Akt pathway
Source: Open Biol. 2015 Sep 23;5(9):150118. doi: 10.1098/rsob.150118 (PMC4593669; doi:10.1098/rsob.150118)
Supplement: p65 NF-kappaB subunit is not involved in NF-kappaB signaling due to L. amazonensis infectio. PI3k/Akt inhibition affects L. amazonensis infection [file rsob150118supp1.doc]

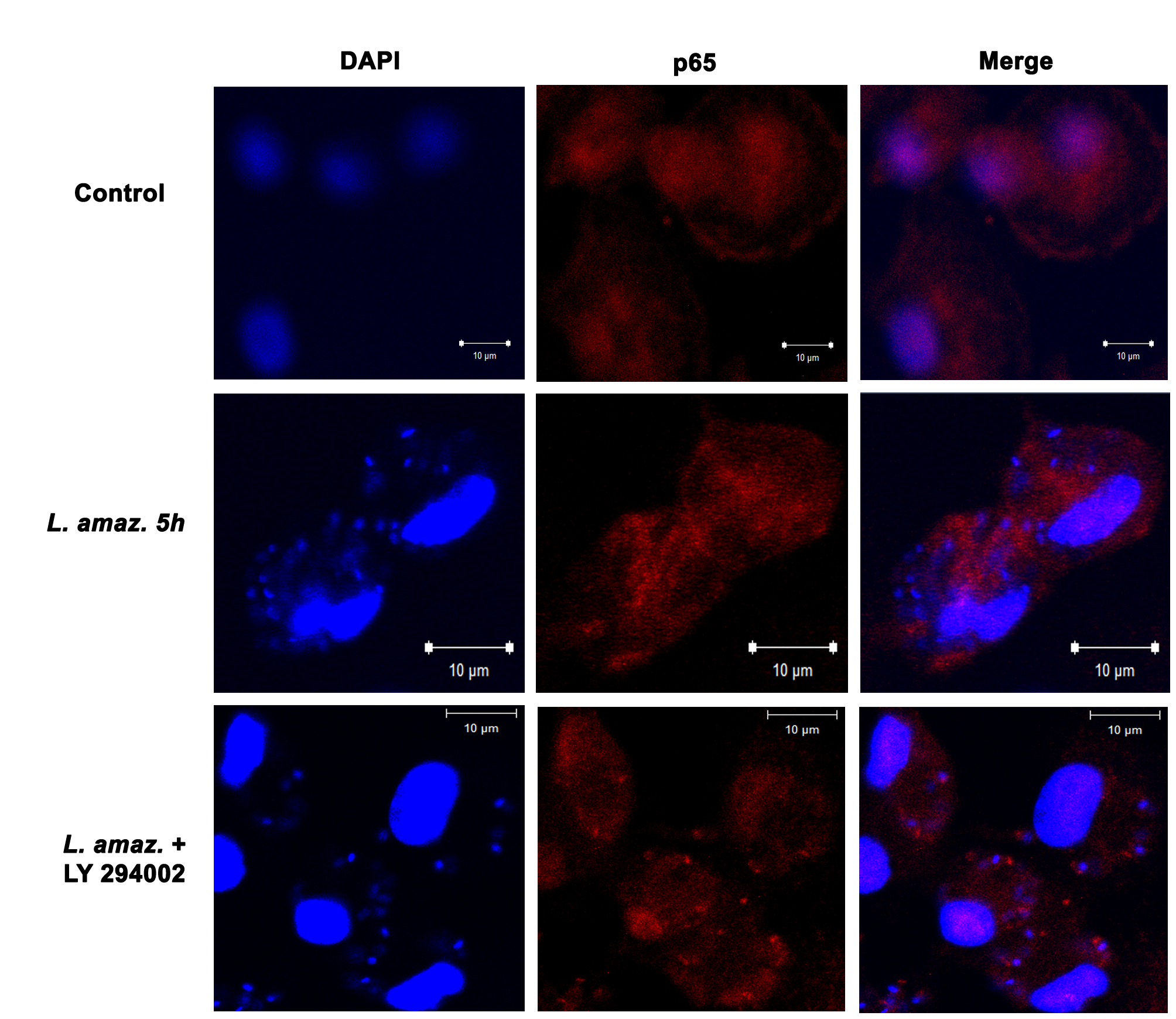


**Supplementary figure 1) p65 NF-κB subunit is not involved in NF-κB signaling due to *L. amazonensis* infection**

Confocal photomicrographs from the infection conditions indicated immunostained with p65 (red) and nuclei stained with DAPI (blue), using 100X objective (bar= 10 μm). Primary human macrophages were infected with *L. amazonensis* for 5 h display citoplasmatic p65 localization.


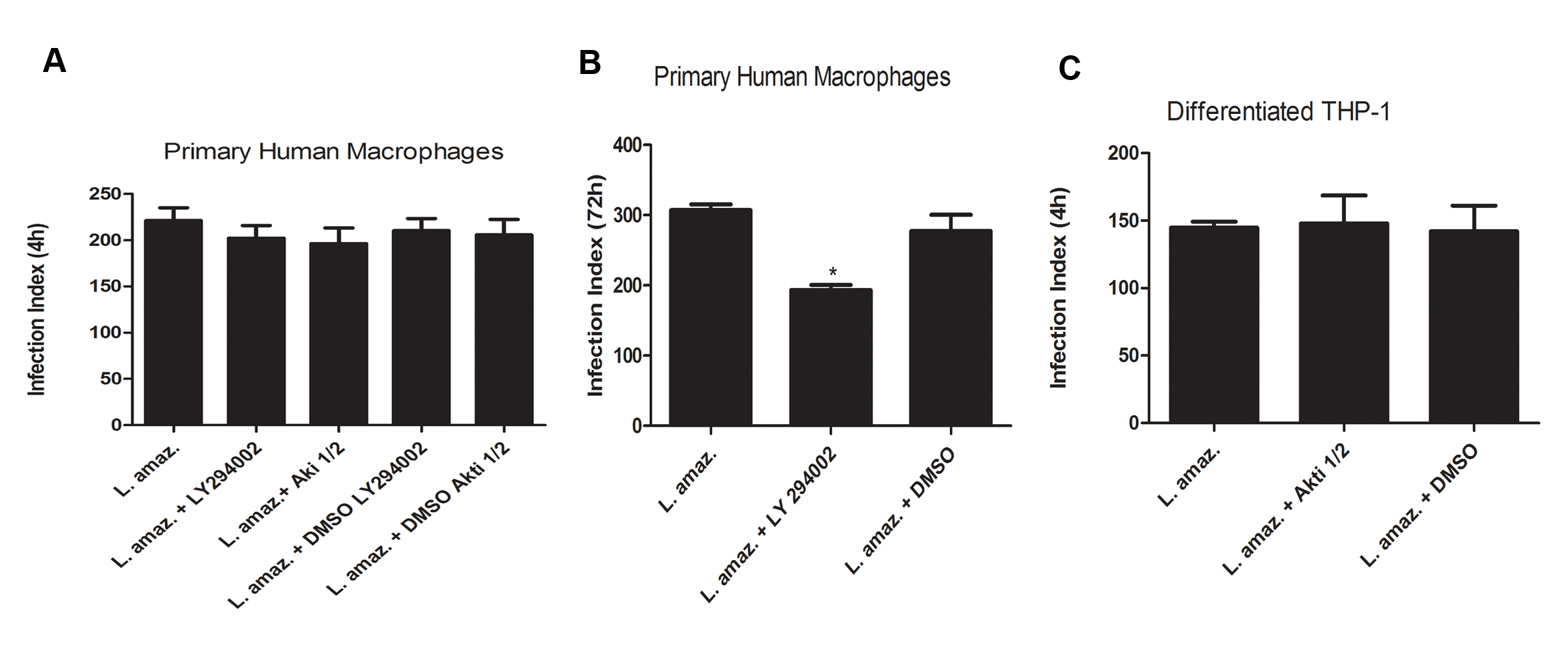


**Supplementary figure 2) PI3k/Akt inhibition affects *L. amazonensis* infection**

Human primary macrophages and differentiated THP1 were infected with promastigote metacyclic of *L. amazonensis* and treated with Akti 1/2 or LY 294002. After 4 or 72h, the infection index was evaluated as described in Materials and Methods. *p<0.05.
